# Clinical Innovation Poster Abstracts

**DOI:** 10.1080/24740527.2022.2088026

**Published:** 2022-08-12

**Covid-19 infection and pain in adolescents with sickle cell disease: A case series**

Doralina Anghelescu^a^, Heidi Meeks^b^, Mike Frett^c^, Latika Puri^d^ and Nicole Alberts^e^

^a^St. Jude Children’s Research Hospital, Pediatric Medicine, Memphis, Tennessee, United States; ^b^St. Jude Children’s Research Hospital, Pediatric Medicine, Memphis, Tennessee, United States; ^c^St. Jude Children’s Research Hospital, Pediatric Medicine, Memphis, Tennessee, United States; ^d^Loma Linda University, Pediatrics, Loma Linda, California, USA; ^e^Concordia University, Psychology, Montreal, Quebec, Canada

**Introduction/Aim**: Pain is the most common clinical manifestation among individuals with sickle cell disease (SCD). Clinically, increases in chronic pain, neuropathic pain, and frequency of acute vaso-occlusive pain have been observed in association with COVID-19 infection among adolescents with SCD. In this case series, we aimed to examine pain complexity in 5 adolescents with SCD who either tested positive for SARS-COV-2 infection or were presumed to have been infected based on detection of antibodies.

**Methods**: Eligible cases were identified through retrospective chart review of cases referred to the St. Jude Children’s Research Hospital Pain Management Service. The criteria utilized to define an increased level of pain complexity after COVID-19 infection included: 1) Increased frequency of acute care visits and/or admissions for pain; 2) New onset chronic pain; 3) New onset neuropathic pain; 4) Escalation in the complexity of pharmacologic therapies; and 5) Increased use of non-pharmacologic interventions.

**Results**: Three of the 5 cases reviewed demonstrated changes in all 5 pain criteria. One case did not display a pattern of increased acute pain episodes frequency and one case did not present a new component of neuropathic pain. However, both of these cases met the remaining criteria used to define increased pain complexity.

**Discussion/Conclusions**: While more research is needed to fully understand the implications of COVID-19 infection on pain in adolescents with SCD, these cases suggest the presence of a complex relationship. Implications of these findings including further investigation of COVID-19 and SCD pain will be discussed.

**The Sensory Evaluation Network: Realigning the role of sensory testing**

Martine Bordeleau^a^, Jan Vollert^b^, Miroslav Backonja^c^, Serge Marchand^d^ and Guillaume Léonard^e^

^a^Université de Sherbrooke, Sherbrooke, Quebec, Canada; ^b^Imperial College London, London, UK; ^c^University of Wisconsin, Madison, USA; ^d^Université de Sherbrooke, Sherbrooke, Quebec, Canada; ^e^Université de Sherbrooke, Sherbrooke, Quebec, Canada

**Introduction/Aim**: Chronic pain is a rising problem in aging societies. Accurate diagnostic methods are required to precisely differentiate pain phenotypes and select the best course of treatment. In clinical and research settings, methods for assessing somatosensory function have included quantitative sensory testing, conditioned pain modulation, nerve conduction studies, evoked potential studies, and others. There are numerous technical variations for each of these approaches, indicating a clear need to align them conceptually.

An online private forum is an interesting method for gathering professional experiences and insights for strategic decisions. The main goal of the Sensory Evaluation Network (SEN) is to bring together international experts in the evaluation of somatic sensory function. Our first goal is to establish a forum of experts to bring forward new concepts, ideas, and recommendations. Our second goal is to build an open, online learning platform for researchers, clinicians, and trainees looking to improve their knowledge and skills about sensory evaluation.

**Methods**: To obtain systematic feedback from SEN members, an iterative approach will be used in conjunction with Slack, an online meeting platform that allows users to view and respond to each other’s comments. Members of the SEN will be asked to respond to open-ended “think aloud” prompts, which will be followed by increasingly specific probes prepared by the moderators. Emerging themes and proposed methodological best practices will be drawn from the online discussion, and the resulting recommendations will be disseminated via peer-reviewed articles and our online learning platform.

**Results**: Not applicable

**Discussion/Conclusions**: Not applicable

**Developing a patient engagement strategy for youth and caregivers in pediatric pain research: Key considerations around equity, diversity, and inclusion**

Yvonne Brandelli^a^, Isabel Jordan^b^, Christine Chambers^c^, Sean Mackinnon^d^, Jennifer Parker^e^, Adam Huber^f^, Jennifer Stinson^g^ and Jennifer Wilson^h^

^a^Dalhousie University, Psychology and Neuroscience, Halifax, Nova Scotia, Canada; ^b^IWK Health Centre, Centre for Pediatric Pain Research, Halifax, Nova Scotia, Canada; ^c^Dalhousie University & IWK Health Centre, Psychology and Neuroscience & Pediatrics, Halifax, Nova Scotia, Canada; ^d^Dalhousie University, Psychology and Neuroscience, Halifax, Nova Scotia, Canada; ^e^IWK Health Centre, Centre for Pediatric Pain Research, Halifax, Nova Scotia, Canada; ^f^Dalhousie University & IWK Health Centre, Pediatrics & Rheumatology, Halifax, Nova Scotia, Canada; ^g^University of Toronto & The Hospital for Sick Children, Lawrence S. Bloomberg Faculty of Nursing & Research Institute, Toronto, Ontario, Canada; ^h^Cassie + Friends, Vancouver, British Columbia, Canada

**Introduction/Aim**: Patient engagement is a growing interest within the field of pediatric pain as the value that patient expertise can contribute to research is increasingly prioritized. Although the inclusion of diverse voices is a foundational principal of patient engagement, little guidance exists on how and where to find such expertise. This methods poster describes our team’s experiences developing a patient engagement strategy to recruit youth with juvenile idiopathic arthritis (JIA) and their caregivers as team members on a research study.

**Methods**: In partnership with Cassie + Friends, a community-based organization, we launched an open call to recruit four youth (13-18 years old) with JIA and/or their caregivers to collaborate on a research study. Advertisements detailing the study topic, eligibility, commitment, and benefits were created through the lens of equity and inclusion (e.g., intentional use of partnership language) and shared on social media (Facebook, Instagram, Twitter). Applicants provided demographic information (e.g., age, gender, disability) and qualitatively shared why this opportunity was of interest. Over 3 weeks, 57 applications were received.

**Results**: In consultation with a strategic lead on patient partnerships (IJ), the following criteria were hierarchically developed to select a diversity of voices to join the team: 1) diverse diagnoses, 2) diverse demographics, and 3) qualitative information.

**Discussion/Conclusions**: Developing patient partnerships is a burgeoning topic. This poster describes the development of a patient engagement strategy to recruit youth and caregiver collaborators. Considerations around the role of equity, diversity, and inclusion in research as well as research ethics will be discussed.

**Improving accessibility to nitrous oxide in a pediatric hospital despite a pandemic**

Evelyne D.Trottier^a^, Marie Joelle Doré-Bergeron^b^, Julie Paquette^c^, Patricia Laforce^d^, Annie Lacroix^e^ and Edith Villeneuve^f^

^a^CHU Sainte-Justine, Pediatric emergency, Montreal, Quebec, Canada; ^b^CHU Sainte Justine, Pediatric, Montreal, Quebec, Canada; ^c^CHU Sainte Justine, Pediatric, Montreal, Quebec, Canada; ^d^CHU Sainte Justine, Pediatric, Montreal, Quebec, Canada; ^e^CHU Sainte Justine, Pediatric, Montreal, Quebec, Canada; ^f^CHU Sainte Justine, Anesthesia, Montreal, Quebec, Canada

**Introduction/Aim**: For some children, distraction, comfort positioning and topical anaesthetic will not provide sufficient peri-procedural comfort and nitrous oxide (N2O) can be a key adjunct. The aim of this quality improvement (QI) initiative was to increase N2O accessibility in a pediatric tertiary care hospital in order to improve procedural pain and distress management.

**Methods**: This QI initiative was supported by the hospital-wide initiative Tout doux, which aims to decrease procedural pain and distress in a pediatric hospital. It includes training the healthcare providers (HP) in the 4Ps approach and a specific training on N2O use. E-learning and formal presentation sessions were available according to HP preferences. Certification with 2 simulated case-scenarios was obtained by all participants prior to supervised first-use with patients.

**Results**: In 2019, the training was first offered on the general wards to all the nurses(59), resulting in different levels of comfort using the technique. The implementation was then modified to focus on ‘N2O champions’. The QI initiative had a pause in March 2020 because of the covid-19 pandemic. A multidisciplinary guideline was created for the use of N2O in the institution in accordance with the new reality: http://www.urgencehsj.ca/protocoles/covid-19-sedation/. Update sessions were offered: http://www.urgencehsj.ca/savoirs/protoxyde-dazote-nitronox/. N2O Champion were trained nurses in the day hospital(18), ED(17), mobile team(7), nursing direction(13) and haematologic team(27) for a total of 141 HPs trained. HP reported use of N2O painful procedures and children with special needs.

**Discussion/Conclusions**: Education initiatives and collaboration successfully increased the use of N2O in a tertiary pediatric hospital despite the arrival of the pandemic.

**Introducing the first Child Life Specialist in an ED for procedural pain and distress management: Successes and challenges**

Evelyne D.Trottier^a^, Kaitlen Gattuso^b^, Marie Joelle Doré-Bergeron^c^, Sarah Loemba^d^, Valérie Leclair^e^, Julie Paquette^f^, Annie Lacroix^g^, Corinne Thériault^h^ and Jocelyn Gravel^i^

^a^CHU Sainte-Justine, Pediatric emergency, Montreal, Quebec, Canada; ^b^CHU Sainte Justine, Pediatric emergency, Montreal, Quebec, Canada; ^c^CHU Sainte Justine, Pediatric, Montreal, Quebec, Canada; ^d^CHU Sainte Justine, Pediatric, Montreal, Quebec, Canada; ^e^CHU Sainte Justine, Pediatric, Montreal, Quebec, Canada; ^f^CHU Sainte Justine, Pediatric, Montreal, Quebec, Canada; ^g^CHU Sainte Justine, Pediatric, Montreal, Quebec, Canada; ^h^CHU Sainte Justine, Pediatric emergency, Montreal, Quebec, Canada; ^i^CHU Sainte-Justine, Pediatric emergency, Montreal, Quebec, Canada

**Introduction/Aim**: Medical procedures can cause pain/distress for children in the emergency department(ED). Non-pharmacological strategies such as preparation, comfort positions, distraction and relaxation techniques can help children cope with procedures. Child Life Specialist(CLS) can facilitate these methods. Objective:To evaluate the impact of introducing a CLS in a pediatric ED for the procedural care of children.

**Methods**: This quality improvement(QI) project was supported by the hospital-wide initiative Tout doux, aiming to alleviate procedural pain/distress across a pediatric hospital. A pilot project involving the introduction of a CLS in the ED was implanted in July2021. To evaluate the impact of pain/distress management techniques, CLS interventions were prospectively recorded to report the strategies used, including non-pharmacological methods, over a 2-month period.

**Results**: Between Sept1st-Nov 1^st^, 2021, the CLS participated in 137 procedures over 40shifts for 108 patients (mean age: 5.5yo[6months-17yo]). Most frequent procedures were 82(60%) blood draws/IV-lines insertion. Parents were present in 132(96%). For 93(68%) procedures, the child/parent had preparation. Distraction was done for all, using active (eg.electronic games 57[42%]), or passive distraction (eg.video 47[34%], music 39[29%]). Deep breathing was utilized in 41(30%). A restrained lying position was used in 61(45%). Topical anesthetic was used for 16/71(23%) children undergoing venous blood draws/IV-line insertions. For 72(53%), procedural pain/distress were considered by the CLS as well managed. For the remaining, in (37/63[59%]), only one strategy was employed.

**Discussion/Conclusions**: Procedures were considered a pain management success in about half of patients. Multimodal pain and distress management techniques and earlier implication of the CLS should be employed to improve this result.

**Within-person analysis of ubrogepant treatment of mild versus moderate-severe headache pain during a phase 3 long-term safety extension trial**

Goran Davidovic^a^, Richard Lipton^b^, David Dodick^c^, Peter Goadsby^d^, Sung Yun Yu^e^, Brittany Jordan^f^, Julia Ma^g^, Janette Contreras-De Lama^h^ and Rami Burstein^i^

^a^AbbVie Inc., Markham, Ontario, Canada; ^b^Albert Einstein College of Medicine, Bronx, New York, USA; ^c^Mayo Clinic, Scottsdale, Arizona, USA; ^d^King’s College Hospital, London, UK; ^e^AbbVie Inc., Madison, New Jersey, USA; ^f^AbbVie Inc., Madison, New Jersey, USA; ^g^AbbVie Inc., Madison, New Jersey, USA; ^h^AbbVie Inc., Irvine, California, USA; ^i^Harvard Medical School, Center for Life Science, Boston, Massachusetts, USA

**Introduction/Aim**: We aimed to assess the efficacy of ubrogepant 50 or 100 mg within participants who treated migraine attacks with mild and moderate/severe pain during a 52-week treatment period. We hypothesized that ubrogepant efficacy within individual participants who treated both mild and moderate/severe pain attacks would be similar to that observed in the previous pooled analysis which demonstrated improved ubrogepant efficacy.

**Methods**: Post-hoc analysis of a subset of the original pooled analysis, where ubrogepant-treated participants with valid efficacy data who treated both at least 3 mild and 3 moderate/severe pain migraine attacks, was performed from phase 3, open-label, long-term safety extension trial. Efficacy measures included pain freedom at two hours after initial dose (2hPF) and absence of migraine-related symptoms. A generalized linear mixed model with binomial distribution and logit link function was applied to assess the treatment effect of ubrogepant at different level of baseline pain intensities.

**Results**: Analysis population included 117 50 mg and 127 100 mg ubrogepant-treated participants. In the 100 mg ubrogepant group: 51.2% of participants with mild pain versus 28.9% with moderate/severe pain (Odds ratio [95% CI] 2.58 [2.24, 2.96]; p<0.001) experienced 2hPF; with absence of photophobia being 65.2% versus 47.5% (2.07 [1.80, 2.37]; p<0.001); absence of phonophobia being 76.6% versus 63.1% (1.91 [1.66, 2.20]; p<0.001); and absence of nausea being 88.0% versus 79.1% (1.94 [1.63, 2.31]; p<0.001), respectively. Results were similar in the 50 mg ubrogepant group.

**Discussion/Conclusions**: These results suggest that ubrogepant is more effective when treating migraine headache while pain intensity is mild versus moderate/severe.

**Determining the Effectiveness of Incorporating Physician Assistants in a Chronic Pain Care Clinic to Improve Access to Care**

Jaclyn De Azevedo^a^ and Deanna Kroetsch^b^

^a^McMaster University Medical Centre, Michael G. Degroote Pain Clinic`, Hamilton, Ontario, Canada; ^b^McMaster University Medical Centre, Michael G Degroote Chronic Pain Clinic, Hamilton, Ontario, Canada

**Introduction/Aim**: The Physician Assistant (PA) profession has continued to be incorporated into the Canadian health care system. PAs help address challenges patients face seeking timely access to health care services. Our goal was to determine if PAs can reduce patient wait times for initial consultation, increase capacity of patients seen both in person and virtually, and assess the efficacy of having multiple PAs.

**Methods**: Reviewed total volume of patients seen at clinic pre-PA vs with PA, number of patients seen by each PA, and average wait time to receive a consultation.

**Results**: The number of new consult and follow up visits have increased from 2,560 in 2014 to 6,911 in 2019 and 8,754 in 2021. This represents a 2.70 & 3.42 fold increase, respectively, since the addition of PAs. The PA’s work collaboratively with 14 physicians on an alternating schedule seeing an average of 35% of all new consultations and 19% of all follow up appointments. Wait times for a new consult have reduced from an average of 2 years in 2014, to 7 months in 2021. In addition to this dramatic reduction in new consult wait time, the physician is now able to see follow up patients much sooner, in person or on a virtual basis.

**Discussion/Conclusions**: Overall, the data supports the value and role of multiple PA’s within the MGD Pain clinic, as this allows a patient to be seen in a timely fashion and also addresses the issue of access to specialized chronic pain care treatment.

**From research to clinical practice: the use of Transcranial Direct Current Stimulation as a new clinical service at “Centre Intégré de Santé et Services Sociaux Abitibi-Témiscamingue” to treat chronic pain**

Rodrigo Deamo Assis^a^, Dat Nhut Nguyen^b^, Marie Philippe Harvey^c^ and Guillaume Leonard^d^

^a^“Centre Intégré de Santé et Services Sociaux Abitibi-Témiscamingue”, Rouyn-Noranda, Quebec, Canada; ^b^“Centre Intégré de Santé et Services Sociaux Abitibi-Témiscamingue”, Rouyn-Noranda, Quebec, Canada; ^c^Université de Sherbrooke, Sherbrooke, Quebec, Canada; ^d^Université de Sherbrooke, Sherbrooke, Quebec, Canada

**Introduction/Aim**: Although tDCS is very known at the research field, it is not used at the clinical practice. Since 2018, the CISSS-AT uses the tDCS to treat chronic pain. Our aim is to describe the effects of tDCS to manage chronic pain in a clinical practice environment.

**Methods**: 5-consecutive-days of active tDCS, 20 minutes combined with mindfulness meditation; anode electrode over the M1 region; cathode electrode over contralateral supraorbital region; and intensity of 2 mA. Inclusion criteria’s: to be a patient of CISSS-AT clinic of pain; to have signs of central sensibilization; and do not progress with the exercise’s program. Exclusion criteria: to have a neurodegenerative disease or a cognitive impairment. The Visual Analog Scale of Pain (VAS), McGill Pain Questionnaire – short version (MPQ) and the Central Sensibilization Inventory (CSI) were administered during 3 periods: first day of tDCS (T1), last day of tDCS (T2) and 1 month after the last day of tDCS (T3). For the analysis, we calculated the medium and the standard deviation.

**Results**: 61 patients made the protocol. Decrease of the score in all the tests were observed between T1/T2 and T1/T3. VAS: T1 6.1 ± 1.7; T2 3.6 ± 1.8 and T3 4.5 ± 1.6. MPQ: T1 21.4 ± 7.8; T2 8.9 ± 5.1 and T3 12.6 ± 5. CSI: T1 55.9 ± 11.2; T2 24.9 ± 9.6 and T3 29.4 ± 9.2. Side effects: fatigue, headache and tingling sensation.

**Discussion/Conclusions**: tDCS seems to be effective to treat chronic pain in the clinical practice environment.

**Chronic Pain and Disability Management in Newfoundland Labrador: A Virtual Stepped Care Approach**

Julie Dwyer^a^ and Heather Foley^b^

^a^University of Edinburgh, St. John’s, Newfoundland & Labrador, Canada; ^b^Memorial University, St. John’s, Newfoundland & Labrador, Canada

**Introduction/Aim**: The Centre for Pain and Disability Management (CPDM) is an interprofessional pain rehabilitation program located in St. John’s, Newfoundland. In March 2020, the CPDM suspended its in-person five-week program due to the COVID-19 lockdown and modified it to virtual delivery incorporating a stepped-care approach. The goal was to provide equitable, uninterrupted access to person-centred, evidence-informed services despite continuously changing public health measures.

**Methods**: The program launched in August 2020 and included changes to the referral screening process. The stepped care model followed a biopsychosocial approach and was comprised of four steps: virtual coaching by a CPDM therapist through a webinar series; virtual coaching by a CPDM therapist through the Therapy Assistance Online pain education modules; virtual participation in the five-week intensive rehabilitation program; and virtual participation in follow-up tailored to individual needs. The CPDM clinicians were responsible for assisting the clients in their set-up and use of the technology required to participate in the program.

**Results**: Since August 2020, there was no interruption in program delivery despite fluctuations in public health guidelines. The wait time to first contact post-referral was reduced from 20-26 months to 12 months. Access barriers associated with travel/accommodations were reduced allowing clients living in rural areas to participate.

**Discussion/Conclusions**: Benefits include more efficient waitlist management, reduced barriers to service access based on residential location, and a clear path towards providing the appropriate service at the aoppriate time. Challenges include increased technical support demands on clinicians, barriers to access based on client technology limitations, and loss of in-person physical assessment.

**Use of trigger point injection for intractable calf pain From Charcot-Marie-Tooth: A case report**

Michael Finnern^a^ and Michelle Poliak-Tunis^b^

^a^University of Wisconsin Hospitals and Clinics, Department of Orthopedics and Rehabilitation, Madison, Wisconsin, USA; ^b^University of Wisconsin Hospitals and Clinics, Department of Orthopedics and Rehabilitation, Madison, Wisconsin, USA

**Introduction/Aim**: Charcot Marie Tooth Disease (CMT) is the most common form of inherited neuromuscular disease and is generally characterized by progressive distal weakness, muscle atrophy, and sensory loss^1^. Muscle cramps have been reported as a significant ailment to quality of life (QOL) in CMT^2^. Studies indicate that patients with subtype CMT1A have an increased frequency of calf cramps which worsen with age, and the resulting calf pain is detrimental to overall QOL in CMT1A^3^. Various treatment methods exist to address this symptom, but little, if any, literature exists regarding the use of trigger point injections (TPI) to address chronic calf pain in patients with CMT.

**Methods**: This case involves a 78-year-old female with a history of CMT who presented with worsening chronic bilateral calf pain. Exhaustive physical therapy, pharmacotherapy, and stretching did not provide adequate relief. The patient was therefore deemed a candidate for TPI, and the more problematic right gastrocnemius was targeted by itself first as a trial. Using a 30-gauge 1/2 inch needle and sterile technique, two trigger points were injected with a total of 1.5mL 1% lidocaine using repeated mechanical lysis. There was reproduction and then cessation of local twitch response. There were no adverse sequelae.

**Results**: One month after the first injection, the patient reported a 50% reduction in pain. She therefore underwent bilateral gastrocnemius TPI with continued monitoring for thus far successful relief of symptoms.

**Discussion/Conclusions**: This report illustrates the use of TPI for the successful treatment of intractable calf pain resulting from CMT.

**Chronic pain management in cancer: An exploratory analysis of electroencephalograph activity during virtual reality pain distraction therapy**

Bernie Garrett^a^, Henry Fu^b^, Gordon Tao^c^, Elliott Cordingley^d^, Zahra Ofoghi^e^, Crystal Sun^f^, Teresa Cheung^g^ and Tarnia Taverner^h^

^a^University of British Columbia, School of Nursing, Vancouver, British Columbia, Canada; ^b^Simon Fraser University, Vancouver, British Columbia, Canada; ^c^University of British Columbia, Vancouver, British Columbia, Canada; ^d^University of British Columbia, Vancouver, British Columbia, Canada; ^e^Simon Fraser University, Vancouver, British Columbia, Canada; ^f^University of British Columbia, Vancouver, British Columbia, Canada; ^g^Simon Fraser University, Vancouver, British Columbia, Canada; ^h^University of British Columbia, Vancouver, British Columbia, Canada

**Introduction/Aim**: This presentation explores the recording and analysis of electroencephalogram data (EEG) during virtual reality (VR) pain distraction therapies in cancer patients with chronic pain associated with cancer and its treatment. Experimental design, recruitment, recording, and analysis of EEG signals and findings will be discussed.

**Methods**: A single-subject design study was used to explore EEG activity during the VR therapy, and pain levels pre and post exposure. Participants were purposively selected, completing or had completed cancer treatment and had ongoing cancer or cancer-treatment related chronic pain. Sixty-four channel EEGs were recorded during an 8-minute pre-exposure rest, 30-minute VR therapy, and 8-minute post-exposure rest. The power of EEG waveforms was compared between each condition using cluster-based permutation testing. A topographic analysis and coherence exploration was performed to identify the variations in power and coherence in different cortices of the brain.

**Results**: A power increase in the beta and gamma bandwidths during the VR therapy was observed with significance (P<.025). Coherence changes during meditation were observed predominantly between the frontal, parietal, and occipital cortices and in the theta, alpha and gamma bands (P<.0025). No significant relationships between pain scores and EEG power variations were observed.

**Discussion/Conclusions**: The study demonstrates specific EEG changes during the VR therapy, and provides novel EEG recording and analysis methods that can be used to investigate neurophysiological changes in VR pain applications. These approaches may guide further studies to explore and identify brain regions and wave bands with respect to VR therapies for patients with cancer related chronic pain.

**Pediatric pain in Alaska: A first look at equitable access in interdisciplinary pain care**

Wendy Gaultney^a^, Ben Ekstrom^b^, Sarah Hanvy^c^, Hailey Lankford^d^ and Charlee Laurie^e^

^a^Neuroversion, Anchorage, Alaska, USA; ^b^Neuroversion, Anchorage, Alaska, USA; ^c^Advanced Physical Therapy, Anchorage, Alaska, USA; ^d^Neuroversion, Anchorage, Alaska, USA; ^e^Neuroversion, Anchorage, Alaska, USA

**Introduction/Aim**: There are approximately 180,000 children and adolescents in Alaska^1^. Given global estimates, approximately 45,000 will meet IASP criteria for chronic pain at some time during childhood/adolescence^2^. Many Alaskan children, especially Alaska Natives, live far from urban centers. Although adult research suggests that pain affects both rural and native populations disproportionately^3^, little is known about the epidemiology of pediatric pain in Alaska. To address the unmet need for high quality pediatric pain care in Alaska, we formed an interdisciplinary program in May 2021. Our team consists of a physician, psychologist, physical therapist, physician’s assistant and medical assistant. This poster aims to describe our program’s reach thus far.

**Methods**: We reviewed demographic information from new patients seen for evaluations to describe who has accessed interdisciplinary pain services.

**Results**: Fifteen patients attended evaluations from June through November. Ages ranged from 8 to 18 years (M=14.15, SD=3.63); 66% identified as female, 20% male and 14% non-binary. Patients identified as white non-Hispanic (66%), Asian Non-Hispanic (13%), white Hispanic (7%), Asian/Black/Hawaiian (7%), Alaska Native (7%). Pain problems included head, abdomen, neck, back, limb and multi-site pain. Patients resided in largely urban areas: Anchorage (47%), the Kenai Peninsula (20%), Matanuska-Susitna (13%), Fairbanks (13%) and Southeast Fairbanks (7%). Patients were evaluated in-person and via telehealth.

**Discussion/Conclusions**: During our first six months we have increased access to interdisciplinary pediatric pain care in Alaska via in-person and telehealth services. As part of our quality improvement efforts we will continue to evaluate and emphasize equity in access to care.

**Unraveling the neuropsychological profile of patients with chronic pain**

Erika Gentile^a^ and Mathieu Roy^b^

^a^McGill University, Psychology, Montreal, Quebec, Canada; ^b^McGill University, Psychology, Montreal, Quebec, Canada

**Introduction/Aim**: Previous studies aiming to objectify cognitive complaints among patients with chronic pain have several methodological limitations (e.g., low sample sizes, heterogeneity of measures) that make it difficult to get a clear portrait of their cognitive impairment. The present study aimed to examine whether chronic pain is predictive of deficits in cognition relative to participants without pain using a large databank in which participants have undergone uniform testing procedures.

**Methods**: 9,339 participants with chronic pain (57% women, mean age: 64.4 years) and 12,620 healthy controls (49% women, mean age: 65.25 years) completed a battery of cognitive tests that were synthesized into the following domains: executive function, learning and memory, processing speed, perceptual reasoning, and fluid reasoning. Sociodemographic and psychological data were accounted for with a psychosocial burden score, calculated from specific questionnaire items.

**Results**: The findings of the present study demonstrate that patient with chronic pain demonstrated poorer performance on tasks of executive functions, fluid reasoning, and memory. Additionally, participants with multiple (two or more) sites of pain performed worse on these measures than those with one pain site or no pain at all.

**Discussion/Conclusions**: The results provide preliminary support for a neurocognitive profile in chronic pain. Such a profile can provide insight into the conceptualization of patient complaints as well as guide differential diagnosis and neuropsychological testing procedures within this population. Further, it can inform treatment plans and targets and be used as a foundation for future investigations of neuropsychological interventions in chronic pain.

**A friend for life: Customized avatar development in an immersive multi-platform virtual environment for children with cancer - Effects on pain, anxiety and satisfaction**

Estelle Guingo^a^, David Paquin^b^, Casey Côtes-Turpin^c^, Christine Genest^d^, Léandra Desjardins^e^, Pascal Bernier^f^, Michel Duval^g^, Cathy Vézina^h^, Marie-France Langlet^i^, Félix Côtes-Charlebois^j^ and Sylvie Le May^k^

^a^Université du Québec en Abitibi-Témiscamingue, Rouyn-Noranda, Quebec, Canada; ^b^Université du Québec en Abitibi-Témiscamingue, Rouyn-Noranda, Quebec, Canada; ^c^Université du Québec en Abitibi-Témiscamingue, Rouyn-Noranda, Quebec, Canada; ^d^Université de Montréal, Montréal, Quebec, Canada; ^e^Centre Hospitalier Universitaire de Sainte-Justine,Montréal, Quebec, Canada; ^f^Centre Hospitalier Universitaire de Sainte-Justine,Montréal, Quebec, Canada; ^g^University Hospital Center Sainte-Justine, Pediatric oncology department, Montreal, Québec, Canada; ^h^Université du Québec en Abitibi-Témicamingue, Rouyn-Noranda, Quebec, Canada; ^i^University Hospital Center Sainte-Justine, Patients-Family-Caregivers partnership office, Montreal, Québec, Canada; ^j^Université du Québec en Abitibi-Témiscamingue, Creation and new medias department, Rouyn-Noranda, Québec, Canada; ^k^Université de Montréal, Montréal, Quebec, Canada

**Introduction/Aim**: Our objective is to study the feasibility, acceptability and effects on pain, anxiety and satisfaction of parents and children undergoing cancer treatment, related to an immersive multi-platform distraction (virtual reality and mobile) based on a customized friendly avatar.

**Methods**: Hospitalized children from 6 to 17 yo. with cancer will be recruited (N=5). Qualitative feedback will be collected from a parent logbook and by semi-structured child-parents interviews. The mobile application will be used as a logbook for children during the trial. A focus group will be organized to collect clinicians’ opinions. We will also measure pain and anxiety using the Numerical Rating Scale and the Child Fear Scale, respectively. Satisfaction will be measured by surveys.Our study follows a co-design approach in art-based research (Research action design). Children will be involved in game’s conception by creating their virtual friend. Game data will be collected to learn about children’s experience.

**Results**: Our preliminary tests are currently running on healthy participants (N=4) following a Think Aloud design approached. The first test provided feedback on how to adapt user’s experience in virtual reality in young children. One of the children (6 yo) expressed difficulties interacting with virtual objects. More results are pending.

**Discussion/Conclusions**: While videogames have previously been tested as therapeutic tools for pain and anxiety management, no studies have evaluated the benefit of a custom avatar-based game for children with cancer. This is an innovative intervention adapted to hospital context.

**Partners in Pain: evaluation of a virtual community engagement group for people living with chronic pain in Saskatchewan**

Cassie Jones^a^, Jessica Jack^b^, Megan Hewson^c^, Alexandria Pavelich^d^, Dr. Colleen Dell^e^, Dr. Karen Lawson^f^, Dr. Pamela Downe^g^, Dr. Krista Baerg^h^, Ross McCreery^i^, Erin Beckwell^j^, Sharon Okeeweehow^k^, Jeannie Coe^l^, Cristina Ugolini^m^, Karen Juckes^n^ and Dr. Susan Tupper^o^

^a^University of Saskatchewan, College of Nursing, Yorkton, Saskatchewan, Canada; ^b^University of Saskatchewan, Archaeology and Anthropology, Saskatoon, Saskatchewan, Canada; ^c^Saskatchewan Health Authority, Regina, Saskatchewan, Canada; ^d^University of Saskatchewan, Sociology, Saskatoon, Saskatchewan, Canada; ^e^University of Saskatchewan, Sociology, Saskatoon, Saskatchewan, Canada; ^f^University of Saskatchewan, Psychology, Saskatoon, Saskatchewan, Canada; ^g^University of Saskatchewan, Archaeology and Anthropology, Saskatoon, Saskatchewan, Canada; ^h^University of Saskatchewan, Pediatrics, Saskatoon, Saskatchewan, Canada; ^i^No Affiliation, Patient Partner, Regina, Saskatchewan, Canada; ^j^University of Regina, Social Work, Saskatoon, Saskatchewan, Canada; ^k^No Affiliation, Patient Partner, Saskatoon, Saskatchewan, Canada; ^l^Saskatchewan Health Authority, Primary Health, Saskatoon, Saskatchewan, Canada; ^m^Saskatchewan Health Authority, Primary Health, Saskatoon, Saskatchewan, Canada; ^n^University of Saskatchewan, College of Nursing, Regina, Saskatchewan, Canada; ^o^Saskatchewan Health Authority, Clinical Excellence, Saskatoon, Saskatchewan, Canada

**Introduction/Aim**: People living with persistent pain report social isolation. The Improving Pain in Saskatchewan (IPSK) research team and the Saskatchewan Pain Society (SaskPain) developed Partners in Pain (PiP), a virtual community support and education group offered twice per month. Results are presented on evaluation responses collected between October 2021 and February 2022.

**Methods**: Each hour-long PiP session consists of brief education, a personal story by someone living with pain, and a facilitated activity (e.g. art project, gentle movement). Participants are adults living with pain, family caregivers, or healthcare providers in Saskatchewan recruited through social media, traditional media, and word of mouth. Participants receive an emailed survey after each session consisting of 18 questions tailored to the session content along with links to resources discussed in the session.

**Results**: Attendance per session averaged 24 participants (85% female). An average of 9 survey responses were received per session (response rate = 37%). 85% agree or strongly agree that they are more aware of how to manage their pain after attending the webinar. 85% intend to use the information learned to support pain management. In response to the patient story: 44% felt less alone, 53% could relate, 31% felt encouraged to connect with others, and 83% agreed or strongly agreed to feeling better connected.

**Discussion/Conclusions**: Although attendance was lower than projected, this may suggest the benefit of smaller scale programs driven by local communities. Participants were predominantly women, suggesting PiP meets the unique needs of this population. Further work is needed to identify needs of other populations.

**IIndigenous Peoples and interprofessional health care providers collaborating to manage chronic pain: Building relationships and learning through the Project ECHO Indigenous Chronic Pain and Substance Use**

Andrew Koscielniak^a^, Donna Garstin^b^, Chris Mushquash^c^, Paul Francis^d^, Ronan Wesley^e^, Teresa Trudeau-Magiskan^f^, Marinna Read^g^, Andrea Furlan^h^ and Patricia Poulin^i^

^a^St. Joseph’s Care Group - Thunder Bay, Mental Health, Thunder Bay, Ontario, Canada; ^b^St. Joseph’s Care Group - Thunder Bay, Mental Health, Thunder Bay, Ontario, Canada; ^c^Lakehead University, Psychology, Thunder Bay, Ontario, Canada; ^d^St. Joseph’s Care Group - Thunder Bay, Indigenous Relations, Thunder Bay, Ontario, Canada; ^e^St. Joseph’s Care Group - Thunder Bay, Indigenous Relations, Thunder Bay, Ontario, Canada; ^f^St. Joseph’s Care Group - Thunder Bay, Indigenous Relations, Thunder Bay, Ontario, Canada; ^g^St. Joseph’s Care Group - Thunder Bay, Mental Health, Thunder Bay, Ontario, Canada; ^h^University of Toronto, Toronto Rehabilitation Institute, Toronto, Ontario, Canada; ^i^University of Ottawa, Department of Anesthesiology and Pain Medicine, Ottawa, Ontario, Canada

**Introduction/Aim**: Chronic pain (CP) disproportionally affects Indigenous Peoples. We describe the development and evaluation of the first *Project Extension for Community Healthcare Outcomes (ECHO) Indigenous Chronic Pain and Substance Use* program, through St. Joseph’s Care Group (SJCG). This program connects community-based health care providers (HCPs) to an interprofessional “hub” via videoconference for lectures and case-based learning to improve culturally safe CP care for Indigenous Peoples.

**Methods**: The first ten months consisted of knowledge exchange and partnership building between ECHO leadership and the Indigenous Health Department at SJCG, Indigenous and non-Indigenous HCPs, Elders, and the *First Nations, Inuit, and Metis Wellness ECHO*. We developed the interprofessional hub and 10-session curriculum (January to March, 2022). Sessions include a 20-minute lecture, and a case discussion guided by the Medicine Wheel. Participants provide practice/demographic characteristics and complete questionnaires including self-efficacy, attitudes and behaviours about CP, facilitators and barriers to implementing learning, along with open feedback.

**Results**: This first pilot includes eighty registrants (26% nurses, 13% social workers, 10% physicians) from 7 provinces. We will summarize participants’ demographic and practice characteristics, pre-post changes on all measures, as well as open feedback. We have identified the need to improve our evaluation framework anchored in Indigenous methodologies to improve cultural safety.

**Discussion/Conclusions**: Supported by the initial positive results, we are developing a culturally safe evaluation and research plan for future ECHOs with the goal of improving HCPs’ experience and ability to provide culturally safe care and ultimately lead to better outcomes for Indigenous Peoples living with CP.

**Improving procedural pain and distress management in a tertiary care centre through a quality improvement initiative: The “Tout doux” project**

Sarah Loemba^a^, Patricia Laforce^b^, Marie-Joëlle Doré-Bergeron^c^, Annie Lacroix^d^, Julie Paquette^e^, Marie-France Langlet^f^, Valérie Leclair^g^, Kaitlen Gattuso^h^, Emilie Trempe^i^ and Evelyne D Trottier^j^

^a^CHU Sainte-Justine, Montreal, Quebec, Canada; ^b^CHU Sainte-Justine, Montreal, Quebec, Canada; ^c^CHU Sainte-Justine, Pediatry, Montreal, Quebec, Canada; ^d^CHU Sainte-Justine, Montreal, Quebec, Canada; ^e^CHU Sainte-Justine, Montreal, Quebec, Canada; ^f^CHU Sainte-Justine, Montreal, Quebec, Canada; ^g^CHU Sainte-Justine, Montreal, Quebec, Canada; ^h^CHU Sainte-Justine, Montreal, Quebec, Canada; ^i^CHU Sainte-Justine, Montreal, Quebec, Canada; ^j^CHU Sainte-Justine, Emergency, Montreal, Quebec, Canada

**Introduction/Aim**: Optimal procedural pain management can decrease children’s distress and pain, increase the child’s collaboration, and improve the family’s health care experience while reducing procedural time and increasing success on the first attempt for healthcare providers (HCP). “Tout doux” is an institutional quality improvement (TD-QI) initiative that aims to improve procedural pain and distress management throughout CHU Sainte-Justine. Aim: To demonstrate the strategic deployment currently being implemented institutionally, to improve procedural pain and distress management to pediatric patients.

**Methods**: With a coordinated structure, TD-QI is organized through eight work teams with different mandates. A pre-established deployment plan has been approved by the hospital’s governance. For each sector, we hold a meeting with the nursing advisors and chief departments to organize the deployment. Afterward, we conduct audits of the procedures. Through an e-learning module or official lectures, HCP received procedural pain training including the “4P” approaches. Field support is offered for 2 months by a pain champion and tools and resources are distributed to HCP. We conduct audits every three months to evaluate the impact of the deployment.

**Results**: So far, 190 HCP have received training, including the hematology-oncology department(169), outpatient pediatric clinic(3+), plastic clinic(14), and collection center(13). The project will be further deployed in the Orthopedics, ENT, ED, and Surgery departments.

**Discussion/Conclusions**: As TD-QI’s initiative to improve procedural pain management, the next step will be to ensure its optimal dissemination through the organization. Moreover, using the CHUSJ’s mandate with SolutionKidsInPain as their Francophone Regional Hub, diffusion through the network is ongoing.

**Providing French-speaking Canadians families with adequate tools and resources on vaccination for their children in the context of a pandemic**

Sarah Loemba^a^, Patricia Laforce^b^, Julie Paquette^c^, Marie-Joëlle Doré-Bergeron^d^, Annie Lacroix^e^, Marie-France Langlet^f^, Valérie Leclair^g^, Kaitlen Gattuso^h^, Emilie Trempe^i^ and Evelyne D Trottier^j^

^a^CHU Sainte-Justine, Montreal, Quebec, Canada; ^b^CHU Sainte-Justine, Montreal, Quebec, Canada; ^c^CHU Sainte-Justine, Montreal, Quebec, Canada; ^d^CHU Sainte-Justine, Pediatry, Montreal, Quebec, Canada; ^e^CHU Sainte-Justine, Montreal, Quebec, Canada; ^f^CHU Sainte-Justine, Montreal, Quebec, Canada; ^g^CHU Sainte-Justine, Montreal, Quebec, Canada; ^h^CHU Sainte-Justine, Montreal, Quebec, Canada; ^i^CHU Sainte-Justine, Montreal, Quebec, Canada; ^j^CHU Sainte-Justine, Emergency, Montreal, Quebec, Canada

**Introduction/Aim**: With the ongoing COVID-19 pandemic, distribution of vaccination resources is important to help protect the population. It is noticed that resources for procedural pain and distress management are not as abundant and diverse for French-speaking knowledge users.

**Methods**: Through their partnership with the organization Solutions for Kids in Pain, the institutional quality improvement initiative *Tout doux* (TD-QI), from CHU Sainte Justine, aims at further improving access to resources for pediatric French-speaking patients and healthcare providers (HCP) on pain and distress prevention and management related to procedures, including vaccination.

**Results**: In summer 2021, TD-QI established to develop a web page with French-written resources for parents and HCP. With frequent demands for getting access to more resources on vaccination, TD-QI decided to produce concise, and accessible tools dedicated to this procedure. This included an infographic handout as well as two educational videos in French intended for patients and families, one of them being specifically made for children. Moreover, an e-learning module was created to help HCP improve their practice of pain and distress management during vaccination.

**Discussion/Conclusions**: The next step will be to ensure optimal dissemination of the tools through the ongoing vaccination campaign.

**Feasibility of implementation of environmental interventions from the CARD (Comfort Ask Relax Distract) system to reduce fear, pain, and fainting during school-based immunizations**

Charlotte Logeman^a^, Anna Taddio^b^, Sarah Fadaleh^c^, Joanne Coldham^d^, Cheri Little^e^, Tracy Samborn^f^, Lucie Bucci^g^, Victoria Gudzak^h^, Meghan McMurty^i^, Joanne Snider^j^, Noni MacDonald^k^ and Cindy Dribnenki^l^

^a^Hospital for Sick Children, Toronto, Ontario, Canada; ^b^Hospital for Sick Children, Leslie Dan Faculty of Pharmacy, Toronto, Ontario, Canada; ^c^Leslie Dan Faculty of Pharmacy, University of Toronto, Toronto, Ontario, Canada; ^d^Alberta Health Services, Calgary, Alberta, Canada; ^e^Alberta Health Services, Calgary, Ontario, Canada; ^f^Alberta Health Services, Calgary, Ontario, Canada; ^g^Immunize Canada, Ottawa, Ontario, Canada; ^h^Leslie Dan Faculty of Pharmacy, University of Toronto, Toronto, Ontario, Canada; ^i^University of Guelph, Department of Psychology, Guelph, Ontario, Canada; ^j^Alberta Health Services, Calgary, Alberta, Canada; ^k^Dalhousie University, Department of Pediatrics, Halifax, Nova Scotia, Canada; ^l^Alberta Health Services, Calgary, Alberta, Canada

**Introduction/Aim**: Environmental factors contribute to students’ fear, pain, and fainting/dizziness during school-based immunizations. The CARD™ (C-comfort, A-ask, R-relax, D-distract) system provides a framework for immunization program delivery that includes environmental interventions (e.g., reducing fear cues, enhancing privacy, and providing separate waiting areas). We evaluated public health staff’s ability to implement these interventions during school-based immunizations.

**Methods**: Four community health centres providing immunization services in Calgary, Alberta implemented CARD across 50 schools with grade 6 and 9 students. Public health staff were educated about environmental interventions ahead of time. Staff reported compliance with the interventions during immunization clinics, including preferred clinic space, private room, separate waiting areas with seating, distraction kits, exercise mat, obscuring fear cues (equipment and individuals getting immunized), and window coverings for clinic doors.

**Results**: Altogether, 62 vaccination clinics were conducted across participating schools. Staff reported high compliance with most interventions: preferred clinic spaces (92%), availability of private rooms (95%), distraction kits (100%), exercise mat (95%), obscuring fear cues (100%), and covering windows (89%). In some cases, however, interventions were deemed inadequate (e.g., private spaces far from the main clinic space). Separate waiting areas with seating were available less frequently (79%) due to physical space limitations.

**Discussion/Conclusions**: Most CARD environmental interventions were feasible to implement across schools. Some interventions, however, were deemed inadequate. More education is recommended to allow staff to more effectively advocate for suitable environmental interventions to ensure that students have positive immunization experiences.

**Choosing care collaboratively: Implementing shared decision making to improve families’ engagement in choosing in-person versus virtual care for youth with chronic pain**

Nicole E MacKenzie^a^ and Kathryn A Birnie^b^

^a^Dalhousie University, Psychology and Neuroscience, Halifax, Nova Scotia, Canada; ^b^University of Calgary, Department of Anesthesiology, Perioperative and Pain Medicine, and Department of Community Health Sciences, Calgary, Alberta, Canada

**Introduction/Aim**: Shared decision making (SDM) is desired by many families, however opportunities to engage in SDM for children’s pain management and care delivery are infrequently made available. The absence of SDM may result in inadequate pain management and lower satisfaction with care; however, engagement in SDM may lead to improved communication between health professionals and families, and improved fit of the decision.

**Methods**: Our team has engaged with diverse youth, families, and health professionals who identified a need for SDM about in-person versus virtual care for pediatric chronic pain. We have conducted a preliminary literature search to identify factors for consideration in SDM for pediatric pain, with conceptual application to deciding about in-person versus virtual care.

**Results**: Identified relevant factors to SDM in pediatric pain include weighing the available evidence-based options, quality and clarity of the evidence available, patient’s and/or parent’s preferences for decision making, decision complexity, and relevant ethical and practical considerations. Our work is identifying current practices and opportunities for decision making for in-person versus virtual care and will determine key features of a decision aid to support this choice.

**Discussion/Conclusions**: Based on these findings, we will create a decision aid that supports an evidence-informed and equitable approach for families and health professionals to reach a shared decision about in-person versus virtual care for pediatric chronic pain. Development and dissemination of this decision aid will empower all families to make a choice that ultimately enhances their engagement with pain management services for the child, resulting in improved pain management.

**Providing specialized chronic pain care support to Primary Care Providers via the “Mobile Clinic”: A preliminary analysis of implementing an evidence-based mobile consultation program at TAPMI**

Ayesha Malik^a^ and Tania Di Renna^b^

^a^Women’s College Hospital, Toronto Academic Pain Medicine Institute (TAPMI), Toronto, Ontario, Canada; ^b^Women’s College Hospital, Toronto Academic Pain Medicine Institute (TAPMI), Toronto, Ontario, Canada

**Introduction/Aim**: Primary Care Providers (PCPs) struggle to care for patients with complex presentations of chronic pain. Complex pain patients who see multiple specialists have common comorbidities such as mental illness, lack of self-management strategies, difficulty accepting prognosis, and high dose opioids. To better support PCPs with such patients, a specialized “Mobile Clinic” was set up as part of a multi-hospital pain program partnership in Toronto (TAPMI). The Mobile Clinic consists of a one-time consultation offered with the PCP, patient, Mobile Clinic staff, and any other specialists willing to attend.

**Methods**: Between November 2020 to October 2021, TAPMI received 43 referrals for complex pain patients who had seen multiple specialists. A pre- and post-questionnaire was sent to the referring PCP before and after attending the Mobile Clinic consultation. The questionnaires gathered data on PCP practice, comfort managing chronic pain and experience with the mobile clinic.

**Results**: The mobile clinic offered a discussion around prognosis, a structured self-management program, and deprescription of high dose opioids. After this, patients were discharged back to PCPs with explicit instruction to promote self-management and decrease number of referrals specifically for pain. All PCPs who attended the clinic felt the clinic was useful in their ongoing management of patients and felt more comfortable managing complex chronic pain in the primary care setting.

**Discussion/Conclusions**: “Mobile clinics” are evidence-based solutions for PCPs struggling to manage patients with complex chronic pain and have shown to increase PCP comfort managing such patients.

**Uncovering medial foot pain caused by tendon intersection syndrome: A case report**

Joseph Anthony Petruccelli^a^ and Chan Gao^b^

^a^Sherbrooke University, Sherbrooke, Quebec, Canada; ^b^Peking University, McGill University, Vanderbilt University, Physical Medicine & Rehabilitation, Montreal, Quebec, Canada

**Introduction/Aim**: Intersection syndrome of the foot is a rare and poorly documented condition characterized by tenosynovitis of the flexor hallucis longus (FHL) resulting from repetitive friction at the crossover of the FHL and the flexor digitorum longus (FDL), known as the knot of Henry, in the plantar midfoot. The objective of this clinical case report is to increase the awareness of this condition when managing chronic foot pain.

**Methods**: An 18-year-old female swimmer presented with constant medial right foot pain, and intermittent redness and swelling on the dorsal medial foot for the past 2 years. The pain is exacerbated by walking more than 15 minutes and is temporarily relieved with ice. She had intermittent numbness, tingling, and cramping affecting the right calf and medial bottom right foot. The physical exam revealed tenderness at the medial right midfoot, with right FHL and FDL weakness, the inability to tiptoe, and single right leg stand. EMG, CT and MRI of the right foot were all negative except for mild thickening of the deltoid ligament.

**Results**: A tibial nerve block at the sustentaculum tali resulted in 70% pain relief for 3 weeks. Then, lateral FHL peri-tendon infiltration with 20 mg of triamcinolone and 1ml of 1% lidocaine at the knot of Henry led to persistent pain relief and the resolution of functional limitations for the past 3 months.

**Discussion/Conclusions**: This presentation sheds light on pedal intersection syndrome as a differential diagnosis for chronic medial foot pain, and its management with ultrasound-guided corticosteroid injection at the knot of Henry.

**Changes in brain GABA levels and improvements in physical functioning following intensive pain rehabilitation in youth with chronic pain**

Taylor Pigott^a^, Allison McPeak^b^, Amanda de Chastelain^c^, Nivez Rasic^d^, Laura Rayner^e^, Melanie Noel^f^, Jillian Vinall Miller (co-senior author)^g^, Ashley Harris (co-senior author)^h^

^a^University of Calgary, Psychology; Biological Sciences, Calgary, Alberta, Canada; ^b^University of Calgary, Medicine, Calgary, Alberta, Canada; ^c^Vi Riddell Children’s Pain & Rehabilitation Centre, Alberta Children’s Hospital, Calgary, Alberta, Canada; ^d^Vi Riddell Children’s Pain & Rehabilitation Centre, Alberta Children’s Hospital, Anesthesiology, Perioperative & Pain Medicine; Child Brain & Mental Health Program, Calgary, Alberta, Canada; ^e^Vi Riddell Children’s Pain & Rehabilitation Centre, Alberta Children’s Hospital, Anesthesiology, Perioperative & Pain Medicine, Calgary, Alberta, Canada; ^f^Vi Riddell Children’s Pain & Rehabilitation Centre, Alberta Children’s Hospital; Owerko Centre, Alberta Children’s Hospital Research Institute; The Mathison Centre for Mental Health Research & Education, Hotchkiss Brain Institute, Psychology; Anesthesiology, Perioperative & Pain Medicine; Child Brain & Mental Health Program; Brain and Behaviour, Calgary, Alberta, Canada; ^g^Vi Riddell Children’s Pain & Rehabilitation Program; Owerko Centre, Alberta Children’s Hospital Research Institute; The Mathison Centre for Mental Health Research & Education, Hotchkiss Brain Institute, Psychology; Anesthesiology, Perioperative & Pain Medicine; Child Brain & Mental Health Program; Brain and Behaviour, Calgary, Alberta, Canada; ^h^Owerko Centre, Alberta Children’s Hospital Research Institute, Radiology, University of Calgary; Child Brain & Mental Health Program; Brain and Behaviour, Calgary, Alberta, Canada

**Introduction/Aim**: A 3-to-6-week multidisciplinary, day-treatment, Intensive Pain Rehabilitation Program (IPRP) was developed at the Alberta Children’s Hospital to help youth with unmanaged chronic pain (pain >3 months) and functional disability. Dysregulation of GABA is thought to play a role in the chronification of pain due to over-excitation of inhibitory brain pathways. We investigated the effect of IPRP on levels of GABA in pain-related brain regions: the anterior cingulate cortex (ACC) and left posterior insula (LPI). We hypothesized that GABA would decrease across IPRP in the ACC and LPI, and this decrease would correlate with improved functioning.

**Methods**: 3T MRI scans were obtained on 23 youth (mean age=16.09±1.40, female=82.6%) at baseline and discharge from IPRP. GABA concentrations were measured using GABA-edited MEGA-PRESS and analyzed using Gannet. At baseline and discharge objective physical measures including the 6-minute walk test were recorded, and patients completed the PROMIS® pain interference questionnaire.

**Results**: Repeated MANOVA revealed a significant decrease in LPI GABA (F=5.374, p=.031), but not ACC GABA (n.s.) after controlling for time between scans. Applying GEE, the decrease in LPI GABA accounted for increased distance in the 6-minute walk test (B=-149.378, p<.001) and decreased pain interference (B=5.744, p=.014).

**Discussion/Conclusions**: LPI is involved in intensity encoding, localization, learning, and memory of painful events. IPRP may have contributed to the normalization of inhibitory tone within this region. Appropriate functioning of the LPI could have contributed to the improvements in physical outcomes pre- to post-IPRP. This research provides objective evidence for IPRP’s role in inhibitory control of pain pathways in youth.

**Family physicians’ adherence to pharmacological recommendations of the clinical pharmacist and the chronic pain anesthesiologist**

Anne-Marie Pinard^a^, Dominique Cronier^b^, Élodie Traverse^c^, Orlane Ballot^d^, Laurence Vezina^e^ and Arianne Jalbert^f^

^a^Centre d’Expertise en Gestion de la Douleur Chronique au CHUL du Chu de Québec - Université Laval.; Centre Interdisciplinaire de Recherche en Réadaptation et Intégration Sociale., Québec, Quebec, Canada; ^b^Centre d’Expertise en Gestion de la Douleur Chronique au CHUL du Chu de Québec - Université Laval., Québec, Quebec, Canada; ^c^Centre d’Expertise en Gestion de la Douleur Chronique au CHUL du Chu de Québec - Université Laval; Centre Interdisciplinaire de Recherche en Réadaptation et Intégration Sociale., Québec, Quebec, Canada; ^d^Centre d’Expertise en Gestion de la Douleur Chronique au CHUL du Chu de Québec - Université Laval; Centre Interdisciplinaire de Recherche en Réadaptation et Intégration Sociale., Québec, Quebec, Canada; ^e^Faculté de pharmacie, Université Laval, Québec, Quebec, Canada; ^f^Faculté de pharmacie, Université Laval, Québec, Quebec, Canada

**Introduction/Aim**: Access to pain clinic services is difficult due to the waiting time and the limited number of centers. The Chu de Québec-Université Laval pain clinic is offering a new type of intervention. First, the pharmacist analyses the pharmaceutical treatment of patients suffering from chronic pain; the anesthesiologist discusses the conclusion with the patient and suggestions are sent to their family physician. This study assesses family physicians’; adherence and satisfaction with pharmaceutical recommendations.

**Methods**: Patients who underwent an evaluative pharmacy consultation validated by an anesthesiologist were included in the study. To evaluate if recommendations were respected, pre- and 6-month post-evaluation drug profiles are obtained from patients’; pharmacy. At the same time, family physicians answered to an online questionnaire to confirm their adherence, clinical impact, and satisfaction with the procedure.

**Results**: Twenty-six patients (± 51.5 years old; 58% women) and thirteen physicians were included. The initial medication for pain treatment was mainly the acetaminophen (65%), opioids (62%) and gabapentinoid (58%). Our recommendations were essentially to cross-over with opioids (60%), discontinuation of gabapentinoids (32%), and initiation of Tramadol/Tapentadol (79%), SNRIs (40%), antipsychotic (86%) and topicals (81%). Reasons for suggestions were: medication required but nor prescribed (40%), lack of efficacy (25%) and adverse effects (16%). 92% of physicians agreed with the recommendations and 100% were satisfied with the procedure. 77% of recommendations had a positive clinical impact.

**Discussion/Conclusions**: Preliminary results show that this type of intervention is beneficial, by reducing waiting time and improving the management of chronic pain by primary care physicians.

**Implementation of a remote, stepped care mental health program for pain and mental health or opioid use concerns at Toronto General Hospital during the COVID-19 pandemic**

P. Maxwell Slepian^a^, Joel Katz^b^, Rachel Siegal^c^, Miki Peer^d^, Aliza Weinrib^e^, Nimish Mittal^f^ and Hance Clarke^g^

^a^Toronto General Hospital, University Health Network, Anesthesia and Pain Management, Toronto, Ontario, Canada; ^b^York University, Psychology, Toronto, Ontario, Canada; ^c^Toronto General Hospital, University Health Network, Department of Anesthesia and Pain Management, Toronto, Ontario, Canada; ^d^Toronto General Hospital, University Health Network, Department of Anesthesia and Pain Management, Toronto, Ontario, Canada; ^e^Toronto General Hospital, University Health Network, Department of Anesthesia and Pain Management, Toronto, Ontario, Canada; ^f^Toronto General Hospital, University Health Network, GoodHope Ehlers Danlos Syndrome Program, Toronto, Ontario, Canada; ^g^Toronto General Hospital, University Health Network, Anesthesia and Pain Management, Toronto, Ontario, Canada

**Introduction/Aim**: With support from the CIHR, we implemented a remote, stepped care mental health program for individuals with chronic pain and mental health or opioid use concerns during the COVID-19 pandemic. The objectives of the program were to leverage an already-in-use chronic pain app (Manage My Pain, MMP) to identify patients at need for mental health care, and provide efficient, virtual interventions for these individuals.

**Methods**: All patients in the GoodHope Ehlers-Danlos Syndrome (EDS) Program and the Transitional Pain Service (TPS) at Toronto General Hospital were first invited to engage with MMP, a self-management app, and complete baseline screening for mental health and opioid use (Step 0). Those scoring above clinical cut-offs were offered a one-time virtual Acceptance and Commitment Therapy workshop (Step 1). Patients who remained above cut-offs after the workshop were invited to participate in a 6 session group program based in Dialectic Behavior Therapy (Step 2).

**Results**: 261 EDS and 127 TPS patients were invited to the program. Of these, 128 EDS and 127 TPS patients registered for MMP. 47 EDS and 33 TPS patients completed screening questionnaires, and 31 EDS and 16 TPS patients scored above the clinical cut-offs and were invited to attend mental health programming.

**Discussion/Conclusions**: We successfully created and implemented a remote, virtually-delivered stepped care mental health program for individuals with chronic pain and mental health or opioid use concerns. However, the majority of patients contacted did engage with the program. Barriers to program uptake and strategies to overcome these barriers will be discussed.

